# Clinical impact of the triple‐layered circular stapler for reducing the anastomotic leakage in rectal cancer surgery: Porcine model and multicenter retrospective cohort analysis

**DOI:** 10.1002/ags3.12516

**Published:** 2021-10-07

**Authors:** Ryota Nakanishi, Yoshiaki Fujimoto, Masahiko Sugiyama, Yuichi Hisamatsu, Tomonori Nakanoko, Koji Ando, Mitsuhiko Ota, Yasue Kimura, Eiji Oki, Tomoharu Yoshizumi

**Affiliations:** ^1^ Department of Surgery and Science Graduate School of Medical Sciences Kyushu University Fukuoka Japan; ^2^ Department of Gastroenterological Surgery National Hospital Organization Kyushu Cancer Center Fukuoka Japan

**Keywords:** anastomotic leak, colorectal surgery, postoperative complications, rectal neoplasms, surgical instruments

## Abstract

**Aim:**

To investigate the impact of the triple‐layered circular stapler compared with the double‐layered circular stapler on anastomotic leakage after rectal cancer surgery.

**Methods:**

The bursting pressure was compared between porcine ileocolic anastomoses created using a double‐ or triple‐layered stapler. We also retrospectively analyzed the incidence of severe anastomotic leakage in 194 patients who underwent colorectal anastomosis using a double‐ or triple‐layered circular stapler during rectal cancer resection performed in two cancer centers between January 2015 and April 2021.

**Results:**

In the porcine model, the bursting pressure was higher in anastomoses created using the triple‐layered stapler than the double‐layered stapler (end‐to‐end anastomosis: 26.4 ± 6.2 mm Hg vs 14.5 ± 4.3 mm Hg, *P* = .0031; side‐to‐side anastomosis: 27.7 ± 5.0 mm Hg vs 18.0 ± 2.9 mm Hg, *P* = .0275). Intersectional leakage occurred in 41% and 83% of anastomoses created using the triple‐ or double‐layered stapler, respectively (*P* = .0821). In the clinical cohort, the double‐ and triple‐layered stapler was used in 153 and 41 patients, respectively. The incidence of anastomotic leakage was lower for anastomoses created using the triple‐layered stapler vs the double‐layered stapler (0.0% vs 5.8%, *P* = .0362). In multivariate analysis, the factors independently associated with a lower incidence of anastomotic leakage were female sex (odds ratio: 0.16, 95% confidence interval: 0.01‐0.90, *P* = .0354) and triple‐layered stapler usage (odds ratio: 0.00, 95% confidence interval: 0.00‐0.96, *P* = .0465).

**Conclusion:**

Anastomoses created using a triple‐layered circular stapler had high bursting pressure, which might contribute to a lower incidence of anastomotic leakage after rectal cancer surgery.

## Graphical abstract

In the porcine model, anastomoses created using the triple‐layered circular stapler had higher bursting pressure than the double‐layered stapler. Furthermore, in the multicenter clinical cohort, the use of the triple‐layered circular stapler was independently associated with the absence of anastomotic leakage.

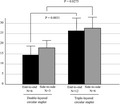

## 1 INTRODUCTION

In rectal cancer surgery, the most common major postoperative complication is anastomotic leakage (AL).^1^, ^2^ A systematic review and meta‐analysis showed that AL after anterior resection for rectal cancer has a negative effect on local recurrence and long‐term survival.^3^, ^4^ AL is also associated with permanent stoma development, fecal incontinence, and reduced sexual activity, which negatively affect patients' long‐term quality of life.^5^, ^6^, ^7^ Furthermore, AL greatly increases the medical costs.^8^ Based on investigations in large patient cohorts, the reported incidence of AL after rectal resection varies from 6.3% to 13.7%.^9^, ^10^, ^11^, ^12^ Some techniques or procedures to prevent AL have been reported, but further improvement is needed.

Recently, the EEA™ Circular Stapler with Tri‐Staple™ Technology (tri‐EEA) (Medtronic Japan, Tokyo, Japan) was released as the first triple‐layered circular stapler, which is expected to decrease the incidence of AL. The tri‐EEA has three rows of staples that vary in height, whereas the previously released EEA™ Circular Stapler with DST Series™ Technology (EEA) (Medtronic Japan) deploys two rows of uniform‐height staples.^13^ In the tri‐EEA, the staples closest to the lumen have the shortest height to provide the greatest occlusion and barrier to leaks and bleeding. The second and third rows, each incrementally higher, contribute strength to the closure line. To the best of our knowledge, no study has evaluated the impact of the tri‐EEA on the anastomotic bursting pressure and incidence of AL in a clinical cohort compared with EEA.

This study was performed to compare the bursting pressure of anastomoses created using the tri‐EEA vs the EEA in a porcine model and to evaluate whether the incidence of AL in a multicenter clinical cohort was lower after rectal cancer surgery using the tri‐EEA than the EEA.

## 2 METHODS

### 2.1 Porcine model

Commercially available small intestine and colon specimens from healthy 6‐month‐old Japanese domestic pigs weighing 100 to 110 kg were used in this study, which was conducted in full accordance with the principles of the Helsinki Institutional Review Board for animal studies. End‐to‐end anastomosis using the double‐stapling technique (DST) was performed as previously described.^14^ The colon was closed and cut with a linear stapler using the Signia™ Stapling System (Tri‐Staple™ 60‐mm purple cartridge; Medtronic Japan). The small intestine was fitted with a 3‐0 Polysorb™ (Medtronic Japan) purse‐string suture around the open stump. The anvil head of the circular stapler (EEA or tri‐EEA) was placed at the edge of the stump, and the anvil shaft was secured. The instrument of the circular stapler was inserted through the open stump of the colon and advanced to the closed stump. The colon wall was penetrated by the center rod of the instrument just beside the colonic closure line. The anvil shaft and the center rod were joined and closed, and the instrument was activated and removed. After anastomosis, it was confirmed that the mucosal layer, muscular layer, and serosa layer were detected without any defect in the entire circumference of both rings that were attached to the instrument.

The side‐to‐side anastomosis was performed as previously described.^14^ The anvil head was inserted through the open stump of the small intestine and advanced to the closed stump. The tip of the anvil head was penetrated through the antimesenteric site 2 cm from the closed stump so that the linear staple line and the anvil head did not overlap. The instrument of the circular stapler was inserted into open stump of the colon and advanced to the closed stump. The center rod was brought through the colonic wall 2 cm from the closed stump so that the linear staple line and the circular staple line did not overlap. The anvil shaft and center rod were then joined and closed, and the instrument was activated and removed. The completion of both rings was confirmed as described for the end‐to‐end anastomosis.

An anastomotic bursting pressure test was performed after the completion of each anastomosis. Both stumps that were not anastomosed were clamped, and a sensor cord was inserted inside the anastomotic site. Air leakage was monitored by placing the specimen in a water‐filled basin and observing the water to detect escaped air bubbles. When air leakage began, the bursting pressure was measured using an electronic manometer (PG‐100N‐102GP; Nidec Copal Electronics, Tokyo, Japan). The leakage site was evaluated to determine whether it was located at the intersection. All experiments were performed in accordance with relevant institutional and national guidelines and regulations for the care and use of animals.

### 2.2 Patients and specimens

A retrospective multicenter study was performed across two comprehensive cancer centers (Kyushu University and National Hospital Organization Kyushu Cancer Center). Of 536 consecutive patients who underwent surgical resection of rectal malignancies and colorectal anastomosis between January 2015 and April 2021, we analyzed 194 patients who underwent colorectal anastomosis using the EEA or the tri‐EEA. We excluded patients in whom other instruments were used for anastomosis, such as the PROXIMATE™ intraluminal stapler (Johnson & Johnson, Tokyo, Japan) or the ECHELON CIRCULAR™ Powered Stapler (Johnson & Johnson) (n = 326), and those who received preoperative chemoradiotherapy (n = 16). The clinicopathological background data were extracted from the database at each hospital. All patients, except those with bowel obstruction, underwent mechanical bowel preparation with magnesium citrate and sennoside on the day before surgery. Surgical procedures were performed or supervised by expert colorectal surgeons qualified by the Endoscopic Surgical Skill Qualification System of Japan,^15^ each of whom had performed more than 200 laparoscopic colorectal operations.

### 2.3 Rectal resection and colorectal anastomosis

The inferior mesenteric artery was clipped and cut at the root, and the left colic artery and inferior mesenteric vein were clipped and cut at the same level. The marginal vessels were preserved. Tumor‐specific mesorectal excision was performed. After clamping the rectum on the anal side of the tumor, the anal canal was washed thoroughly through the anus. The rectum was cut using the Signia™ Stapling System (Tri‐Staple™ 60‐mm purple cartridge). After removing the specimen through the abdominal incision, the anastomosis was performed. End‐to‐end anastomosis was performed using the DST as previously described.^16^ Side‐to‐side anastomosis was performed using a previously described method.^17^ We previously reported that the side‐to‐side anastomosis was a safe and useful procedure in anastomosis after rectal resection.^18^ In this study, the type of anastomosis was selected at the surgeon's discretion, and there was no specific indication for each procedure. If the surgeon intended to perform side‐to‐side anastomosis for very low anterior resection, side‐to‐end anastomosis was sometimes selected because the stump of the rectum was too short to anastomose to the side wall. Therefore, side‐to‐end anastomoses were performed with intersection of the circular and linear staple lines of the remnant rectum. After division of the patients according to whether such intersections were present, we included side‐to‐end anastomosis in the end‐to‐end anastomosis group. An air leak test was performed after each anastomosis, and it was confirmed that no anastomoses had an air leak. Reinforcement was performed at the surgeon's discretion regardless of the leak test result or the anastomosis type. The reinforcement was not always performed at the intersection; some surgeons performed it only at the non‐intersection site. A closed drain was placed behind the anastomotic site in all cases. A transanal drainage tube was placed when considered necessary by the surgeon.

### 2.4 Postoperative management

Oral intake started on postoperative day 3, and the transanal tube was removed on the same day. The intra‐abdominal drain was removed on postoperative day 4. Postoperative complications that occurred during hospitalization and within 90 days after surgery were extracted and classified using the Clavien‐Dindo (CD) system. If patients developed postoperative complications such as fever, abdominal and/or pelvic pain, or elevated inflammatory markers, radiological examinations (computed tomography [CT] and/or transanal enema examination) were performed. AL was diagnosed when a communication between the intra‐ and extraluminal compartments caused by a defect of the intestinal wall integrity at the anastomosis was detected by CT and/or transanal enema examination.^19^ In this study, we focused on AL requiring active therapeutic interventional drainage, transanal drainage, or reoperation (CD grade ≥3). The site of leakage was determined by identification of the defect on CT or endoscopy. If AL was detected, reoperation was performed in patients who had no covering stoma, and interventional drainage or transanal drainage was performed in patients who had a covering stoma. We considered reoperation if the infection could not be controlled by drainage. In patients who underwent covering ileostomy, AL was examined not only during hospitalization and within 90 days after surgery but also after stoma closure (however, no patients in this study developed AL after stoma closure).

### 2.5 Statistical analysis

Continuous variables were analyzed using the Mann‐Whitney U‐test, while categorical valuables were analyzed with Pearson's χ^2^ test or Fisher's exact test, as appropriate. A *P* value of <.05 was considered significant. Statistical analysis was performed using JMP® software version 10.0.2 (SAS Institute).

## 3 RESULTS

### 3.1 Impact of the triple‐layered circular stapler on anastomotic bursting pressure in a porcine model

**FIGURE 1:**
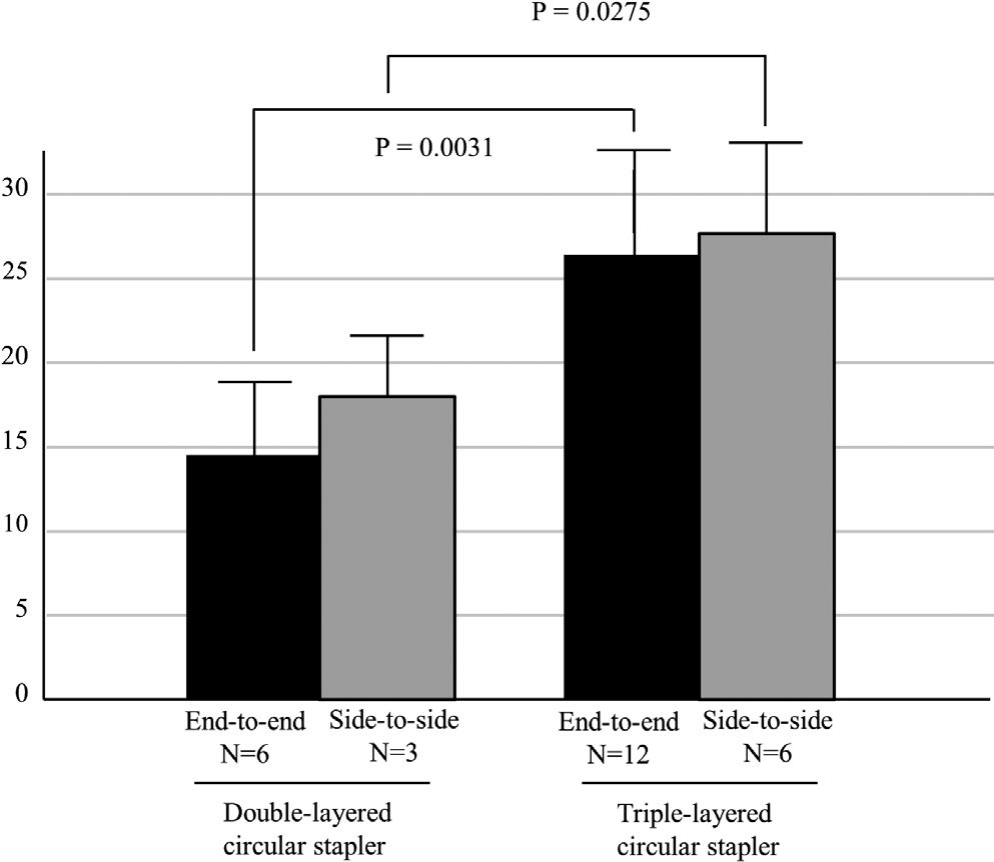
Bursting pressure of anastomoses created using the double‐ or triple‐layered circular stapler. Black bars: end‐to‐end anastomoses; grey bars: side‐to‐side anastomoses

The bursting pressure was measured in a total of 27 anastomoses. End‐to‐end anastomosis (using the DST) was performed six times using the EEA and 12 times using the tri‐EEA. Side‐to‐side anastomosis was performed three times using the EEA and six times using the tri‐EEA. As shown in Figure 1, the bursting pressure was significantly higher in anastomoses created using the tri‐EEA than in those created using the EEA, regardless of the type of anastomosis (end‐to‐end anastomosis: EEA 14.5 ± 4.3 mm Hg vs tri‐EEA 26.4 ± 6.2 mm Hg, *P* =.0031; side‐to‐side anastomosis: EEA 18.0 ± 2.9 mm Hg vs tri‐EEA 27.7 ± 5.0 mm Hg, *P* =.0275). Among the end‐to‐end anastomoses, the leakage was located at the intersection site in five of six anastomoses created using the EEA (83%) and in five of 12 anastomoses created using the tri‐EEA (41%); however, this difference was not significant (*P* =.0821).

### 3.2 Patient characteristics and short‐term outcomes

The patient characteristics are summarized in Table 1. The median age was 66 years, 55% were male, and the median body mass index was 21.9 kg/m^2^. One hundred and eighty‐eight patients had rectal cancer, five had neuroendocrine tumors, and one had an endometrial stromal sarcoma. Among the total cohort, the EEA was used in 153 patients (EEA group) and the tri‐EEA was used in 41 patients (tri‐EEA group). The prevalence of more advanced rectal cancer (stage III‐IV) was higher in the tri‐EEA group than in the EEA group (20% vs 1%, *P* <.0001). There were no significant differences between the two groups in other characteristics, including age (*P* =.6976), sex (*P* =.8912), body mass index (*P* =.9763), history of diabetes mellitus (*P* =.2169), preoperative chemotherapy (*P* =.3988), tumor location (*P* =.4058), and maximum tumor diameter (*P* =.6131).

**TABLE 1:** Patients' background

Characteristics	Total N = 194	EEA group N = 153	tri‐EEA group N = 41	*P* value
Age (years), median (IQR)	66 (56.75‐71.25)	66 (56.5‐71)	67 (56.5‐73)	.6976
Gender, No.(%)				
Male	107 (55)	84 (55)	23 (56)	.8912
Female	87 (45)	69 (45)	18 (44)	
Body mass index (kg/m^2^), median (IQR)	21.9 (19.6‐24.7)	21.8 (19.7‐24.5)	22.1 (19.4‐24.9)	.9763
Diabetes mellitus yes, No. (%)	12 (6)	11 (7)	1 (2)	.2169
Preoperative chemotherapy yes, No. (%)	13 (7)	9 (6)	4 (10)	.3988
Location, No. (%)				
High rectum	85 (44)	67 (44)	18 (44)	.4058
Middle rectum	83 (43)	68 (44)	15 (37)	
Low rectum	26 (13)	18 (12)	8 (20)	
Pathological stage^a^, No. (%)				
I	58 (31)	49 (33)	9 (23)	<.0001
II	53 (28)	41 (28)	12 (30)	
III	68 (37)	57 (39)	11 (28)	
IV	9 (5)	1 (1)	8 (20)	
Maximum tumor diameter (mm), median (IQR)	35 (25‐55)	35 (25‐53)	40 (29.5‐57.5)	.6131
Histologic grade, No. (%)				
Well/moderately differentiated	187 (96)	147 (96)	40 (98)	.7565
Poorly differentiated	1 (1)	1 (1)	0 (0)	
Others^b^	6 (3)	5 (3)	1 (2)	

Abbreviations: BMI: body mass index; EEA, EEA™ Circular Stapler with DST Series™ Technology; IQR, interquartile range; Tri‐EEA, EEA™ Circular Stapler with Tri‐Staple™ Technology.

^a^ Only for rectal cancer patients.

^b^ Five neuroendocrine tumor and one endometrial stromal sarcoma.

The intraoperative characteristics and short‐term outcomes are summarized in Table 2. Compared with the EEA group, the tri‐EEA group had a longer operation time (278 minutes vs 227 minutes, *P* =.0042), lower prevalence of laparoscopic surgery (0% vs 7%, *P* =.0270), higher prevalence of side‐to‐side anastomosis (17% vs 6%, *P* =.0328), and lower prevalence of 25‐mm circular stapler usage (0% vs 64%, *P* <.0001). There were no significant differences between the two groups in intraoperative characteristics, such as the prevalence of low anterior resection (61% in the EEA group vs 51% in the tri‐EEA group, *P* =.2396) and the use of a transanal tube (86% in the EEA group vs 95% in the tri‐EEA group, *P* =.0897). The two groups had similar short‐term outcomes, including the postoperative hospital stay (14 days in the EEA group vs 14 days in the tri‐EEA group, *P* =.4757) and incidence of all postoperative complications (CD grade ≥3) (8% in the EEA group vs 2% in the tri‐EEA group, *P* =.1731). The incidence of AL (CD grade ≥3) was significantly lower in the tri‐EEA group than in the EEA group (0.0% vs 5.8%, *P* =.0362).

**TABLE 2:** Intraoperative characteristics and short‐term outcomes

Characteristics	Total N = 194	EEA group N = 153	tri‐EEA group N = 41	*P* value
Duration of operation (min), median (IQR)	237.5 (191.5‐301.75)	227 (186‐298)	278 (220‐341)	.0042
Bleeding (g), median (IQR)	15 (8‐30)	15 (10‐ 30)	15 (5.5‐33)	.6967
Intraoperative transfusion, yes, No. (%)	3 (2)	2(1)	1(2)	.6218
Procedure, No. (%)				
Open	10 (5)	10 (7)	0 (0)	.0270
Laparoscopy	184 (95)	143 (93)	41 (100)	
Surgery, No. (%)				
Anterior resection	79 (41)	59 (39)	20 (49)	.2396
Low anterior resection	115 (59)	94 (61)	21 (51)	
Covering stoma, yes, No. (%)	47 (24)	34 (22)	13 (32)	.2181
Type of anastomosis, No. (%)				
End‐to‐end/Side‐to‐end	178 (92)	144 (94)	34 (83)	.0328
Side‐to‐side	16 (8)	9 (6)	7 (17)	
Size of circular stapler (mm), No. (%)				
25	99 (51)	98 (64)	0 (0)	<.0001
28	95 (49)	55 (36)	41 (100)	
Transanal tube, yes, No. (%)	171 (88)	132 (86)	39 (95)	.0897
Postoperative hospital stay (day), median (IQR)	14 (11‐19)	14 (11‐19)	14 (11.25‐24)	.4757
Postoperative complication (CDC≥3), No. (%)				
None	181 (93)	141 (92)	40 (98)	.1731
Present	13 (7)	12 (8)	1 (2)	
Anastomotic leakage (CDC≥3), No. (%)				
None	185 (95)	144 (94)	41 (100)	.0362
Present	9 (5)	9 (6)	0 (0)	

Abbreviations: CDC, Clavien‐Dindo Classification; EEA, EEA™ Circular Stapler with DST Series™ Technology; IQR, interquartile range; Tri‐EEA, EEA™ Circular Stapler with Tri‐Staple™ Technology.

### 3.3 Risk factors for anastomotic leakage

The results of the univariate and multivariate analyses are described in Table 3. In univariate analysis, the factors significantly correlated with AL were male sex (*P* =.0247), low anterior resection (*P* =.0444), and the use of the EEA (*P* =.0362). In multivariate analysis adjusted for these factors, the use of the tri‐EEA for anastomosis was independently associated with the absence of AL (hazard ratio [HR] [95% confidence interval (CI)]; 0.00 [0.00‐0.96], *P* =.0465), while male sex was independently associated with AL (HR [95% CI]; 6.40 [1.12‐120.90], *P* =.0354).

**TABLE 3:** Univariate and multivariate analysis for anastomotic leakage

Variable	Univariate analysis		Multivariate analysis	
	HR (95%CI)	*P* value	HR (95%CI)	*P* value
Sex				
Male vs Female	6.95 (1.24‐130.22)	.0247	6.40 (1.12‐120.90)	.0354
Age				
≤65 year vs >65 years	0.86 (0.21‐3.36)	.8297		
BMI				
≤25 kg/m^2^ vs >25 kg/m^2^	0.57 (0.14‐2.79)	.4543		
Location				
High rectum vs middle/low rectum	0.35 (0.05‐1.50)	.1659		
Pathological Stage^a^				
I‐II vs III‐IV	0.55 (0.13‐2.13)	.3769		
Maximum tumor diameter				
≤50 mm vs >50 mm	0.40 (0.08‐1.86)	.2371		
Surgery				
Anterior resection vs low anterior resection	0.17 (0.01‐0.96)	.0444	4.68 (0.11‐1.24)	.0911
Covering stoma				
No vs yes	0.62 (0.16‐3.05)	.5281		
Type of anastomosis				
Side‐to‐side vs end‐to‐end/side‐to‐end	0.00 (0.00‐5.44)	.2076		
Size of circular stapler				
25 vs 28	0.47 (0.10‐1.85)	.2869		
Stapler, No. (%)				
Tri‐EEA vs EEA	0.00 (0.00‐0.86)	.0362	0.00 (0.00‐0.96)	.0465

Abbreviations: BMI, body mass index; CI, confidence interval; EEA, EEA™ Circular Stapler with DST Series™ Technology; HR, hazard ratio; Tri‐EEA, EEA™ Circular Stapler with Tri‐Staple™ Technology.

^a^ Only for rectal cancer.

### 3.4 Details of patients who developed anastomotic leakage

The details of the nine patients with AL are summarized in Table 4. Eight of nine patients were male, and eight had undergone low anterior resection. Three patients had a covering stoma after low anterior resection. All nine patients had a transanal tube. The anastomosis had been performed in an end‐to‐end fashion with the EEA in all nine patients. The leak was located at the intersection site in six patients and a site other than the intersection in three patients.

**TABLE 4:** Details of patients with anastomotic leakage

No.	Sex	Age	BMI	Location	Preoperative chemotherapy	Pathological Stage	Surgery	Covering stoma	Type of anastomosis	Transanal tube	EEA/tri‐EEA	Site of leakage
1	F	24	14.5	Middle rectum	None	IIIB	LAR	None	End‐to‐end	Present	EEA	Not intersection
2	M	87	20.5	Middle rectum	None	IIIB	LAR	None	End‐to‐end	Present	EEA	Intersection
3	M	64	29.0	Middle rectum	None	IIIA	LAR	Present	End‐to‐end	Present	EEA	Intersection
4	M	62	21.6	Upper rectum	None	IIIB	LAR	None	End‐to‐end	Present	EEA	Intersection
5	M	81	24.4	Middle rectum	None	II	LAR	Present	End‐to‐end	Present	EEA	Intersection
6	M	61	28.5	Middle rectum	None	II	LAR	None	End‐to‐end	Present	EEA	Intersection
7	M	75	20.9	Lower rectum	None	I	LAR	None	End‐to‐end	Present	EEA	Not intersection
8	M	67	27.1	Middle rectum	None	II	LAR	Present	End‐to‐end	Present	EEA	Not intersection
9	M	69	19.6	Upper rectum	None	IIIA	AR	None	End‐to‐end	Present	EEA	Intersection

Abbreviations: AR, anterior resection; BMI, body mass index; EEA, EEA™ Circular Stapler with DST Series™ Technology; F, female; LAR, low anterior resection; M, male; Tri‐EEA, EEA™ Circular Stapler with Tri‐Staple™ Technology.

## 4 DISCUSSION

In this porcine model study and the retrospective clinical cohort analysis, we evaluated the impact of the tri‐EEA on the anastomotic strength compared with the previously used EEA. In the porcine model, the anastomoses created using the tri‐EEA had significantly higher bursting pressure than those created using the EEA. Furthermore, in the clinical cohort, the patients in whom the tri‐EEA was used had a significantly lower incidence of AL than those in whom the EEA was used. Use of the tri‐EEA was independently associated with the absence of AL. The present study is the first to demonstrate that triple‐layered anastomosis was superior to double‐layered anastomosis in patients who underwent rectal resection for rectal malignancies.

Previous studies have shown that the intrarectal pressure measured using high‐resolution manometry is 80 mm Hg during simulated defecation and up to 200 mm Hg during squeezing.^20^, ^21^ In addition, the peak intrarectal pressure is higher in males than in females, which might contribute to male sex being a reported risk factor for AL.^11^, ^21^, ^22^ Although these intrarectal pressures are much higher than the anastomotic bursting pressure in our porcine model, the anastomotic bursting pressure in patients who undergo low anterior resection reportedly becomes much higher at 3‐6 months after surgery.^23^ Because high intrarectal pressure during defecation can lead to AL, we usually advise patients to maintain soft stool and avoid excess force during defecation for at least 1‐2 months after surgery. This gap between the intrarectal pressure and the anastomotic bursting pressure is effectively bridged by the placement of a transanal tube and reinforcing sutures, especially in patients with risk factors such as a short distance from the anastomosis to the anus, male sex, and advanced tumor stage.^24^, ^25^ Our study suggests that the higher anastomotic bursting pressure resulting from the use of the tri‐EEA may also be effective in preventing AL.

Other than anastomotic strength and intrarectal pressure, it has been reported that the blood flow and tension at the anastomotic site are important factors related to AL.^26^, ^27^, ^28^, ^29^ A recently proposed technology with which to evaluate the blood flow is indocyanine green fluorescence‐guided visualization, and the clinical significance of this method has been reported.^29^, ^30^ Some studies have shown that high ligation of the inferior mesenteric artery is associated with a higher risk of AL, although this finding is controversial.^31^, ^32^ We do not routinely use indocyanine green fluorescence‐guided visualization, and we clip the inferior mesenteric artery at the root, unless the patient has severe arteriosclerosis on CT, because low ligation sometimes leads to tension at the anastomotic site. Selective mobilization of the splenic flexure is an established method for reducing the tension at the anastomotic site.^26^, ^27^ We usually perform splenic flexure mobilization when there seems to be tension at the anastomotic site. As a result, our overall incidence of AL was 4.6% (9/194), which is lower than that reported in previous studies with large patient cohorts.^9^, ^10^, ^11^, ^12^ However, some previous single‐institutional retrospective studies have reported very low incidences of AL^22^, ^33^; these institutions would have undertaken their own preventative measures to achieve these low incidences. Because AL is caused by multiple factors, every conceivable precautionary measure should be undertaken to reduce the incidence of AL.

End‐to‐end anastomosis (using the DST) is often used for anastomosis after rectal resection. Endoscopic evaluation of patients with AL after anastomosis using the DST has shown that AL often occurs at the intersection of the anastomosis lines of the circular stapler and linear stapler.^14^ Our porcine model study also showed that five of six anastomoses created with the EEA were ruptured at the intersection. In our clinical cohort study, the intersection was ruptured in six of nine patients with AL, and the EEA had been used in all nine of these patients. This finding indicates the presence of a structural weakness at the intersection of anastomoses created with the EEA via the DST. In contrast, less than half of the anastomoses created with the tri‐EEA in our porcine model were ruptured at the intersection. Although the difference in the incidence of rupture at the intersection between the EEA and tri‐EEA groups was not significant, our results might suggest that the tri‐EEA reduces the vulnerability of the intersection. In our porcine model, the anastomoses created with the tri‐EEA had a significantly higher bursting pressure than those created with the EEA, even in side‐to‐side anastomoses that have no intersection. This implies that the use of triple‐layered stapler leads to higher bursting pressure at the circular anastomosis line than the use of the double‐layered stapler. Side‐to‐side anastomosis reportedly produces a superior bursting pressure than that produced by end‐to‐end anastomosis.^18^ Although it is possible that the use of the side‐to‐side anastomosis technique affected the incidence of AL in the present study, the univariate and multivariate analyses did not prove that side‐to‐side anastomosis had a significant impact on the development of AL. Finally, we observed no AL in any patients in the tri‐EEA group. This suggests that AL after colorectal anastomosis with the tri‐EEA might be rare in appropriately managed cases.

The operation time was longer in the tri‐EEA than in the EEA group. However, in the multivariate analysis adjusted for the factors significantly correlated with the operation time in the univariate analysis, a longer operation time (>300 minutes) was significantly associated with the tumor location (Rb vs RS/Ra; HR, 2.38; 95% CI, 1.01‐5.67; *P* =.0498), but not male sex (*P* =.2316) or the device used for the anastomosis (*P* =.4050) (data not shown). This analysis showed no direct relationship between the device used and the operation time.

Our study has some limitations. First, the animal experiments on anastomosis strength were not intracorporeal, and were performed using the colon and small intestine of pigs. We chose to perform extracorporeal experiment so not to be influenced by other factors than the devise (the tri‐EEA or the EEA), such as the preparation status, the blood flow, or the wall thickness of the intestine. Regarding the wall thickness, the rectum usually lacks serosa and has thick muscle layers. Therefore, our animal model did not specifically reflect the actual situation in clinical practice. However, the aim of our animal study was to compare the strength of anastomoses created using the tri‐EEA vs the EEA, and we successfully proved the superiority of anastomoses created using the tri‐EEA. Second, the sample size was relatively small. Although our multicenter study analyzed about 200 patients with rectal cancer, only nine patients had AL. Third, our study is subject to the selection bias inherent in observational retrospective studies. Neoadjuvant chemoradiotherapy is used to reduce the incidence of local recurrence, but it may cause tissue edema, fibrosis, and even necrosis.^34^ There was a meta‐analysis examining the influence of the neoadjuvant chemoradiotherapy to AL, and the authors concluded that neoadjuvant radiotherapy did not increase the incidence of AL.^35^ However, the *P* value was marginal (Odds ratio = 1.24, 95%CI; 0.97‐1.58, *P* =.08), and we thought it was still controversial. So, we excluded the patients who underwent neoadjuvant chemoradiotherapy. The impact of the tri‐EEA on the anastomosis strength in patients who undergo neoadjuvant chemoradiation therapy warrants further investigation. As shown in Tables 1 and 2, the tri‐EEA group had higher prevalence of more advanced rectal cancer, laparoscopic surgery, side‐to‐side anastomosis, and use of the 28‐mm circular stapler. Although advanced tumor stage is reportedly a risk factor for AL,^11^ the tri‐EEA group had a lower incidence of AL. In this study, nine patients had stage IV rectal cancer (Table 1). All of these patients underwent total mesorectal excision with D3 lymph node dissection, and we do not believe that it affected the outcome. Because only 10 patients underwent open surgery, and because all patients who had AL underwent laparoscopic surgery, we could not statistically analyze the impact of laparoscopic surgery on our results. The incidence of AL was associated with the use of the tri‐EEA, even when open surgery was excluded (0.0% in the tri‐EEA group vs 6.3% in the EEA group, *P* =.0307). We used the tri‐EEA with a circular stapler size of 28 mm, while the EEA was used with 25‐, 28‐, and 31‐mm circular staplers; therefore, the size of the circular stapler was 28 mm in all patients in the tri‐EEA group. None of these factors were risk factors for AL in our univariate and multivariate analyses. Further prospective studies are necessary to confirm the clinical significance of anastomosis using the tri‐EEA.

## 5 CONCLUSIONS

This animal model and multicenter retrospective study revealed that anastomoses created with the tri‐EEA had higher bursting pressure than those created with the EEA, and that anastomoses created with the tri‐EEA were associated with a lower incidence of AL. Our study indicated that the use of the tri‐EEA leads to safer rectal surgery, suggesting that the indications for covering stomas might need to be reevaluated.

## AUTHOR CONTRIBUTIONS

Each person listed as an author or coauthor meets all four criteria listed in the Author Guideline of Annals of Gastroenterological Surgery.

## DISCLOSURE

Conflict of Interest: The authors declare no conflicts of interest for this article.

The protocol for this research project has been approved by a suitably constituted ethics committee of each institution (committee of Kyushu University, Approval No. 2019‐209; committee of National Hospital Organization Kyushu Cancer Center, Approval No. 2019‐67), and it conforms to the provisions of the Declaration of Helsinki. Informed consent was obtained from each patient.

Our experimental porcine model was established by purchasing commercially available small intestine and colon specimens; it was not performed as a live animal experiment.

## References

[1] Hallbook O , Sjodahl R . Anastomotic leakage and functional outcome after anterior resection of the rectum. Br J Surg. 1996;83(1):60–2. 8653367 10.1002/bjs.1800830119

[2] Rullier E , Laurent C , Garrelon JL , Michel P , Saric J , Parneix M . Risk factors for anastomotic leakage after resection of rectal cancer. Br J Surg. 1998;85(3):355–8. 9529492 10.1046/j.1365-2168.1998.00615.x

[3] Ma L , Pang X , Ji G , Sun H , Fan Q , Ma C . The impact of anastomotic leakage on oncology after curative anterior resection for rectal cancer: a systematic review and meta‐analysis. Medicine (Baltimore). 2020;99(37):e22139. 32925766 10.1097/MD.0000000000022139 PMC7489661

[4] Yang J , Chen Q , Jindou L , Cheng Y . The influence of anastomotic leakage for rectal cancer oncologic outcome: a systematic review and meta‐analysis. J Surg Oncol. 2020;121(8):1283–97. 32243581 10.1002/jso.25921

[5] Kverneng Hultberg D , Svensson J , Jutesten H , Rutegård J , Matthiessen P , Lydrup M‐L , et al. The impact of anastomotic leakage on long‐term function after anterior resection for rectal cancer. Dis Colon Rectum. 2020;63(5):619–28. 32032197 10.1097/DCR.0000000000001613

[6] Marinatou A , Theodoropoulos GE , Karanika S , Karantanos T , Siakavellas S , Spyropoulos BG , et al. Do anastomotic leaks impair postoperative health‐related quality of life after rectal cancer surgery? A case‐matched study. Dis Colon Rectum. 2014;57(2):158–66. 24401876 10.1097/DCR.0000000000000040

[7] Midura EF , Hanseman D , Davis BR , Atkinson SJ , Abbott DE , Shah SA , et al. Risk factors and consequences of anastomotic leak after colectomy: a national analysis. Dis Colon Rectum. 2015;58(3):333–8. 25664712 10.1097/DCR.0000000000000249

[8] La Regina D , Di Giuseppe M , Lucchelli M , Saporito A , Boni L , Efthymiou C , et al. Financial impact of anastomotic leakage in colorectal surgery. J Gastrointest Surg. 2019;23(3):580–6. 30215201 10.1007/s11605-018-3954-z

[9] Paun BC , Cassie S , MacLean AR , Dixon E , Buie WD . Postoperative complications following surgery for rectal cancer. Ann Surg. 2010;251(5):807–18. 20395841 10.1097/SLA.0b013e3181dae4ed

[10] Kang CY , Halabi WJ , Chaudhry OO , Nguyen V , Pigazzi A , Carmichael JC , et al. Risk factors for anastomotic leakage after anterior resection for rectal cancer. JAMA Surg. 2013;148(1):65–71. 22986932 10.1001/2013.jamasurg.2

[11] Park JS , Choi G‐S , Kim SH , Kim HR , Kim NK , Lee KY , et al. Multicenter analysis of risk factors for anastomotic leakage after laparoscopic rectal cancer excision: the Korean laparoscopic colorectal surgery study group. Ann Surg. 2013;257(4):665–71. 23333881 10.1097/SLA.0b013e31827b8ed9

[12] Watanabe T , Miyata H , Konno H , Kawai K , Ishihara S , Sunami E , et al. Prediction model for complications after low anterior resection based on data from 33,411 Japanese patients included in the National Clinical Database. Surgery. 2017;161(6):1597–608. 28153378 10.1016/j.surg.2016.12.011

[13] Medtronic.com. [internet]. Available from: https://www.medtronic.com/covidien/en‐us/products/surgical‐stapling/eea‐circular‐stapler.html

[14] Ikeda T , Kumashiro R , Taketani K , Ando K , Kimura Y , Saeki H , et al. Endoscopic evaluation of clinical colorectal anastomotic leakage. J Surg Res. 2015;193(1):126–34. 25103641 10.1016/j.jss.2014.07.009

[15] Akagi T , Endo H , Inomata M , Yamamoto H , Mori T , Kojima K , et al. Clinical impact of Endoscopic Surgical Skill Qualification System (ESSQS) by Japan Society for Endoscopic Surgery (JSES) for laparoscopic distal gastrectomy and low anterior resection based on the National Clinical Database (NCD) registry. Ann Gastroenterol Surg. 2020;4(6):721–34. 33319163 10.1002/ags3.12384 PMC7726689

[16] Kuroyanagi H , Akiyoshi T , Oya M , Fujimoto Y , Ueno M , Yamaguchi T , et al. Laparoscopic‐assisted anterior resection with double‐stapling technique anastomosis: safe and feasible for lower rectal cancer? Surg Endosc. 2009;23(10):2197–202. 19116740 10.1007/s00464-008-0260-y

[17] Oki E , Ando K , Saeki H , Nakashima Y , Kimura Y , Hiyoshi Y , et al. The use of a circular side stapling technique in laparoscopic low anterior resection for rectal cancer: experience of 30 serial cases. Int Surg. 2015;100(6):979–83. 25590136 10.9738/INTSURG-D-14-00202.1 PMC4587526

[18] Ando K , Kuriyama N , Fujimoto Y , Jogo T , Hokonohara K , Hu Q , et al. New anastomosis technique to prevent anastomotic leakage in laparoscopic anterior resection for rectal cancer, especially upper rectal cancer. In Vivo. 2020;34(6):3533–8. 33144464 10.21873/invivo.12195 PMC7811646

[19] Rahbari NN , Weitz J , Hohenberger W , Heald RJ , Moran B , Ulrich A , et al. Definition and grading of anastomotic leakage following anterior resection of the rectum: a proposal by the International Study Group of Rectal Cancer. Surgery. 2010;147(3):339–51. 20004450 10.1016/j.surg.2009.10.012

[20] Dinning PG , Carrington EV , Scott SM . The use of colonic and anorectal high‐resolution manometry and its place in clinical work and in research. Neurogastroenterol Motil. 2015;27(12):1693–708. 26224550 10.1111/nmo.12632

[21] Attari A , Chey WD , Baker JR , Ashton‐Miller JA . Comparison of anorectal function measured using wearable digital manometry and a high resolution manometry system. PLoS One. 2020;15(9):e0228761. 32991595 10.1371/journal.pone.0228761 PMC7523952

[22] Akiyoshi T , Ueno M , Fukunaga Y , Nagayama S , Fujimoto Y , Konishi T , et al. Incidence of and risk factors for anastomotic leakage after laparoscopic anterior resection with intracorporeal rectal transection and double‐stapling technique anastomosis for rectal cancer. Am J Surg. 2011;202(3):259–64. 21871980 10.1016/j.amjsurg.2010.11.014

[23] Dulskas A , Samalavicius NE . Usefulness of anorectal manometry for diagnosing continence problems after a low anterior resection. Ann Coloproctol. 2016;32(3):101–4. 27437391 10.3393/ac.2016.32.3.101 PMC4942524

[24] Choy KT , Yang TWW , Heriot A , Warrier SK , Kong JC . Does rectal tube/transanal stent placement after an anterior resection for rectal cancer reduce anastomotic leak? A systematic review and meta‐analysis. Int J Colorectal Dis. 2021;36(6):1123–32. 33515307 10.1007/s00384-021-03851-8

[25] Maeda K , Nagahara H , Shibutani M , Ohtani H , Sakurai K , Toyokawa T , et al. Efficacy of intracorporeal reinforcing sutures for anastomotic leakage after laparoscopic surgery for rectal cancer. Surg Endosc. 2015;29(12):3535–42. 25673349 10.1007/s00464-015-4104-2

[26] Kennedy R , Jenkins I , Finan PJ . Controversial topics in surgery: splenic flexure mobilisation for anterior resection performed for sigmoid and rectal cancer. Ann R Coll Surg Engl. 2008;90(8):638–42. 18990277 10.1308/003588408X358774 PMC2727804

[27] Marsden MR , Conti JA , Zeidan S , Flashman KG , Khan JS , O’Leary DP , et al. The selective use of splenic flexure mobilization is safe in both laparoscopic and open anterior resections. Colorectal Dis. 2012;14(10):1255–61. 22188371 10.1111/j.1463-1318.2011.02927.x

[28] You X , Liu Q , Wu J , Wang Y , Huang C , cao G , et al. High versus low ligation of inferior mesenteric artery during laparoscopic radical resection of rectal cancer: a retrospective cohort study. Medicine (Baltimore). 2020;99(12):e19437. 32195939 10.1097/MD.0000000000019437 PMC7220455

[29] Yanagita T , Hara M , Osaga S , Nakai N , Maeda Y , Shiga K , et al. Efficacy of intraoperative ICG fluorescence imaging evaluation for preventing anastomotic leakage after left‐sided colon or rectal cancer surgery: a propensity score‐matched analysis. Surg Endosc. 2021;35(5):2373–85. 33495878 10.1007/s00464-020-08230-y

[30] Watanabe J , Ishibe A , Suwa Y , Suwa H , Ota M , Kunisaki C , et al. Indocyanine green fluorescence imaging to reduce the risk of anastomotic leakage in laparoscopic low anterior resection for rectal cancer: a propensity score‐matched cohort study. Surg Endosc. 2020;34(1):202–8. 30877565 10.1007/s00464-019-06751-9

[31] Kang J , Choi G‐S , Oh JH , Kim NK , Park JS , Kim MJ , et al. Multicenter analysis of long‐term oncologic impact of anastomotic leakage after laparoscopic total mesorectal excision: the Korean laparoscopic colorectal surgery study group. Medicine (Baltimore). 2015;94(29):e1202. 26200636 10.1097/MD.0000000000001202 PMC4603022

[32] Hajibandeh S , Hajibandeh S , Maw A . Meta‐analysis and trial sequential analysis of randomized controlled trials comparing high and low ligation of the inferior mesenteric artery in rectal cancer surgery. Dis Colon Rectum. 2020;63(7):988–99. 32243350 10.1097/DCR.0000000000001693

[33] Yamaguchi T , Kinugasa Y , Shiomi A , Sato S , Yamakawa Y , Kagawa H , et al. Learning curve for robotic‐assisted surgery for rectal cancer: use of the cumulative sum method. Surg Endosc. 2015;29(7):1679–85. 25277477 10.1007/s00464-014-3855-5

[34] Geisler D , Marks J , Marks G . Laparoscopic colorectal surgery in the irradiated pelvis. Am J Surg. 2004;188(3):267–70. 15450832 10.1016/j.amjsurg.2004.04.007

[35] Hu MH , Huang RK , Zhao RS , Yang KL , Wang H . Does neoadjuvant therapy increase the incidence of anastomotic leakage after anterior resection for mid and low rectal cancer? A systematic review and meta‐analysis. Colorectal Dis. 2017;19(1):16–26. 27321374 10.1111/codi.13424

